# Genome-wide association for sarcoidosis identifies novel risk loci and genetic heritability in African and European ancestries: a meta-analysis from the Finngen, Million Veteran Program, UK Biobank, and Biobank Japan datasets

**DOI:** 10.1186/s13023-025-04097-1

**Published:** 2025-12-29

**Authors:** Andrea Ricci, Federica Andolfi, Daniele Sabbatini, Filippo Gozzi, Giada Di Betto, Paolo Ventura, Elena Buzzetti, Antonello Pietrangelo, Enrico Clini, Roberto Tonelli, Dario Andrisani, Brent Julius de Guzman Marinduque, Elisa Bergamini, Chiara Vecchi, Elena Pegoraro, Dario Gregori, Elena Corradini, Stefania Cerri

**Affiliations:** 1 Centre for Genomic Medicine and Rare Diseases, Internal Medicine Unit, Department of Medical and Surgical Sciences, University Hospital of Modena - Policlinico, University of Modena and Reggio Emilia, Via del Pozzo 71,41121, Modena, Italy; 2 Internal Medicine Unit, University Hospital of Modena - Policlinico, University of Modena and Reggio Emilia, Modena, Italy; 3 Centre for Rare Lung Diseases, Pneumology Unit, Department of Medical and Surgical Sciences, University Hospital of Modena - Policlinico, ERN Lung, University of Modena and Reggio Emilia,Via del Pozzo 71, 41121, Modena, Italy; 4 Department of Neurosciences Dns, University of Padova, Padova, Italy; 5 Unit of Biostatistics, Epidemiology and Public Health, Department of Cardiac, Thoracic, Vascular Sciences, and Public Health, University of Padova, Padova, Italy; 6 School of Medicine and Surgery, University of Modena and Reggio Emilia, Modena, Italy

**Keywords:** Sarcoidosis, GWAS, Meta-analysis, Rare diseases, UK Biobank, FinnGen, Million veteran program, Biobank Japan

## Abstract

**Introduction:**

Sarcoidosis is an inflammatory disease driven by immune-mediated mechanisms, characterized by the formation of epithelioid cell granulomas and a wide range of clinical manifestations. Its phenotype is the result of a complex interplay of genetic and environmental factors, the precise roles and interactions of which remain poorly defined.

**Aim:**

To identify candidate genes and risk loci associated with sarcoidosis from large population datasets. To estimate the genetic heritability of the phenotype in selected ancestries.

**Population and methods:**

Public summary statistics from the FinnGen release 12 (European ancestry), pan UK BioBank Project (UKBB - European and African ancestry), Million Veteran Program (MVP - European and African ancestry), and Japan BioBank (East Asian ancestry) were included for European, African and multi-ancestry meta-analysis through sample size-based analysis. Novel risk loci and single nucleotide polymorphisms (SNPs) significantly associated with the disease were critically reviewed on the basis of the available literature. For each risk locus, SNPs highly correlated with the lead SNP were selected based on Combined Annotation Dependent Depletion (CADD) scores. Genetic heritability (h^2^) scores were obtained through ancestry-specific linkage-disequilibrium score calculation.

**Result:**

Overall 9659 cases (7559 European, 1880 African, 220 East Asian) and 1,665,804 controls (1,361,726 European, 126,411 African, 177,667 East Asian) were analysed. Nineteen and two risk loci were identified in European and African ancestry, respectively; h2 scores were 0.25 (European) and 0.19 (African). Candidate non-MHC genes for further explorations through functional studies included IL23R, PUS10, ACOXL, PLCL1, FAM117B, BMPR2, PPARG, ESYT2, ANXA11, CCDC88B, ATXN2, CCL24, RP11−540O11.1, HOMER2, CD19, UBASH3A, RNF215, and others. Interferon gamma signaling, meiotic recombination/condensation of prophase chromosomes, and DNA methylation were the most enriched gene sets in European and multi-ancestry meta-analysis. Multi-ancestry meta-analysis was confronted with FinnGen+UKBB+MVP meta-analysis (released by FinnGen freeze 12) yielding consistent results (18 risk loci identified)

**Conclusion:**

Nineteen and two risk loci were significantly associated with sarcoidosis for European and African ancestries, respectively. Moderate genetic heritability was observed for both ancestries. A set of significantly associated non-MHC genes and SNPs was obtained to investigate functional validation. Although further studies are warranted, epigenetic alterations may contribute to the risk of developing sarcoidosis

**Supplementary information:**

The online version contains supplementary material available at 10.1186/s13023-025-04097-1.

## Introduction

Sarcoidosis is an inflammatory disease driven by immune-mediated mechanisms, characterized by the formation of epithelioid cell granulomas and a wide range of clinical manifestations [1]. Although the disease occurs worldwide, its prevalence and clinical features have been shown to differ across ethnic groups [2–4]. This variability, along with familial clustering and an increased risk in relatives, underscores its multifactorial nature, reflecting the complex interplay of genetic and environmental factors, the precise roles and interactions of which remain poorly defined [5].

The identification of genetic variants through genome-wide association studies (GWAS), whole exome sequencing (WES), and whole-genome sequencing (WGS) have been conducted with the aim of uncovering the contribution of genetic components to the development of sarcoidosis [6]. These studies have not only confirmed associations with major histocompatibility complex (MHC) class II genes, particularly *HLA-DRB1* and *BTNL2* variants within the HLA class II region, but have also led to the identification of several additional genetic loci/genes that may confer susceptibility to sarcoidosis [7, 8].

The growing availability of data from large population-based GWAS datasets has boosted the potential to identifying new risk loci, as well as corroborating the significance of those already reported in the literature. Moreover, analysis from large multi-ethnic populations can provide additional evidence for the correlation between SNP expression and ethnic variability, given a particular phenotype. In this study, we conducted a meta-analysis of the publicly available summary statistics of four genome-wide association studies (GWAS) for sarcoidosis, including results from European (EUR), African (AFR), and East Asian (EAS) ancestries. Through bioinformatic analyses and critical comparison with the available literature, we sought to identify novel ancestry-specific risk loci, estimate genetic heritability of the disease, and highlight possible metabolic pathways of interest for functional validation.

## Methods

### Datasets and patient population

The latest public updates of the following datasets were accessed:

- FinnGen freeze 12 [9]: https://www.finngen.fi/en/access_results, phenotype SARCOIDOSIS (released on 4 November 2024); the FinnGen study is a large-scale genomics initiative that has analyzed over 500,000 Finnish biobank samples and correlated genetic variation with health data to understand disease mechanisms and predispositions; the project is a collaboration between research organisations and biobanks within Finland and international industry partners https://www.finngen.fi/en;
- Pan-UK Biobank project [10] https://pan.ukbb.broadinstitute.org/, phenotype “D86 sarcoidosis” (last recorded update 16 March 2023); the UK Biobank is a large-scale biomedical database and research initiative that has recruited around 500,000 individuals aged 40 to 69 years and collected extensive genetic and phenotypic information https://www.ukbiobank.ac.uk/;
- Million Veteran Program (MVP) [11] (last recorded update 22 August 2023): Phecodes Phe 697.EUR.GIA (European) and Phe 697.AFR.GIA (African American); the MVP is an ongoing prospective cohort study and biobank in the Department of Veterans Affairs Healthcare System designed to study genetic influences on health and disease among veterans https://www.ncbi.nlm.nih. gov/projects/gap/cgi-bin/study.cgi?study_id = phs001672.v12.p1;
- Biobank Japan (BBJ) https://pheweb.jp/, summary statistics available from [12] (published on 30 September 2021), phenotype sarcoidosis; BioBank Japan is a disease-oriented biobank that receives biological samples and clinical information from participating patients diagnosed with target diseases through cooperative medical institutions nationwide and stores such samples and information https://biobankjp.org/en/.

For each dataset, the following ancestry-specific samples size were reported:

- Finngen SARCOIDOSIS, r0.12, 5411 cases, 492,311 controls;
- Million Veteran Program, EUR ancestry 1509 cases, 449,523 controls;
- Million Veteran Program, AFR ancestry 1827 cases, 119,828 controls;
- UK Biobank (pan-UKBB), EUR ancestry, 639 cases, 419,892 controls;
- UK Biobank (pan-UKBB), AFR ancestry, 53 cases, 6583 controls;
- Japan Biobank, EAS ancestry, 220 cases, 177,667 controls.

All datasets adopted the most recent GRCh38 assembly of human genome except BBJ, which followed the GRCh37 assembly. Therefore, chromosome coordinates in the BBJ dataset were substituted with coordinates from the UKBB reference dataset through rsIDs cross-referencing (R script available in Supplementary data). Prior to performing meta-analysis, all summary statistics were individually replicated. The BBJ, MVP and meta-analysis datasets lacked nearest gene specifications, so annotation through rsIDs cross-referencing from the UKBB reference dataset was performed (R script available in Supplementary data).

### Meta-analyses

Dataset handling, SNP filtering, and plotting of Manhattan and qq plots was performed through R version 4.4.1 [13], using the following packages: parallel, vroom, data.table, dplyr, BiocManager, Biostrings, SeqArray, ggmanh, SeqArray, farver, labeling, readr, knitr, kableExtra. R markdown html reports for the main analyses are available in supplementary data.

Sample size-based meta-analysis of summary statistics from EUR, AFR, and multi-ancestry were performed using METAL [14]. In all analyses, correction for genomic inflation *λ* was applied. A *p*- value < 5 × 10^−8^ was required to assess statistical significance.

Prior to meta-analysis, the datasets were filtered according to a common set of SNPs. AFR-specific meta- analysis included 1880 cases, 126,411 controls; 21,388,456 common SNPs were analysed. EUR-specific meta-analysis included 7559 cases, 1,361,726 controls; 10,776,775 common SNPs were analysed. Multi- ancestry meta-analysis included 9659 cases, 1,665,804 controls; 5,586,486 common SNPs were analysed. A similar multi-ancestry meta-analysis (with no EAS ancestry) was issued with the FinnGen r0.12 freeze and can be accessed at https://mvp-ukbb.finngen.fi/pheno/D3_SARCOIDOSIS.

### Genetic heritability estimation

Genetic heritability (h^2^) was estimated by means of linkage disequilibrium (LD) score regression through the LDSC tool [15, 16]. EUR and AFR LD scores were made available by the pan-UKBB project website [10].

### Risk loci annotation

Risk loci plotting and annotation was performed with Functional Mapping and Annotation of GWAS (FUMA GWAS) [17]; ANNOVAR [18] and Combined Annotation-Dependent Depletion (CADD) [19] were used for functional annotation. FUMA allowed also to perform tissue expression (TEA) and gene- set enrichment analysis (GSEA) through MAGMA [20] and the GENE2FUNC option, based on the Genotype-Tissue Expression (GTEx) project, version 8 [21, 22] for expression quantitative trait locus (eQTL) estimations. Among the available datasets, REACTOME [23] was used for GSEA. The MHC region was excluded from all analyses except GSEA.

Plink 1.9 [24, 25] with GRCh38-based 1000 Genomes (1000 G) Project Phase 3 reference panel [26, 27], was used to clump variants according to linkage disequilibrium (r^2^ > 0.6). Plink and the 1000 G reference (EUR and AFR ancestries) were also used by FUMA to calculate r^2^ and minor allele frequency estimations for risk loci plotting and annotation.

For each locus in EUR ancestry meta-analysis, SNPs used for mapping were manually selected based on their CADD score ( > 12.37). Literature search was conducted to critically assess the significance of the identified genes.

## Result

Overall 9659 cases (7559 European, 1880 African, 220 East Asian) and 1,665,804 controls (1,361,726 European, 126,411 African, 177,667 East Asian) were analysed.

### Risk loci identification

#### EUR ancestry

**Fig. 1:**
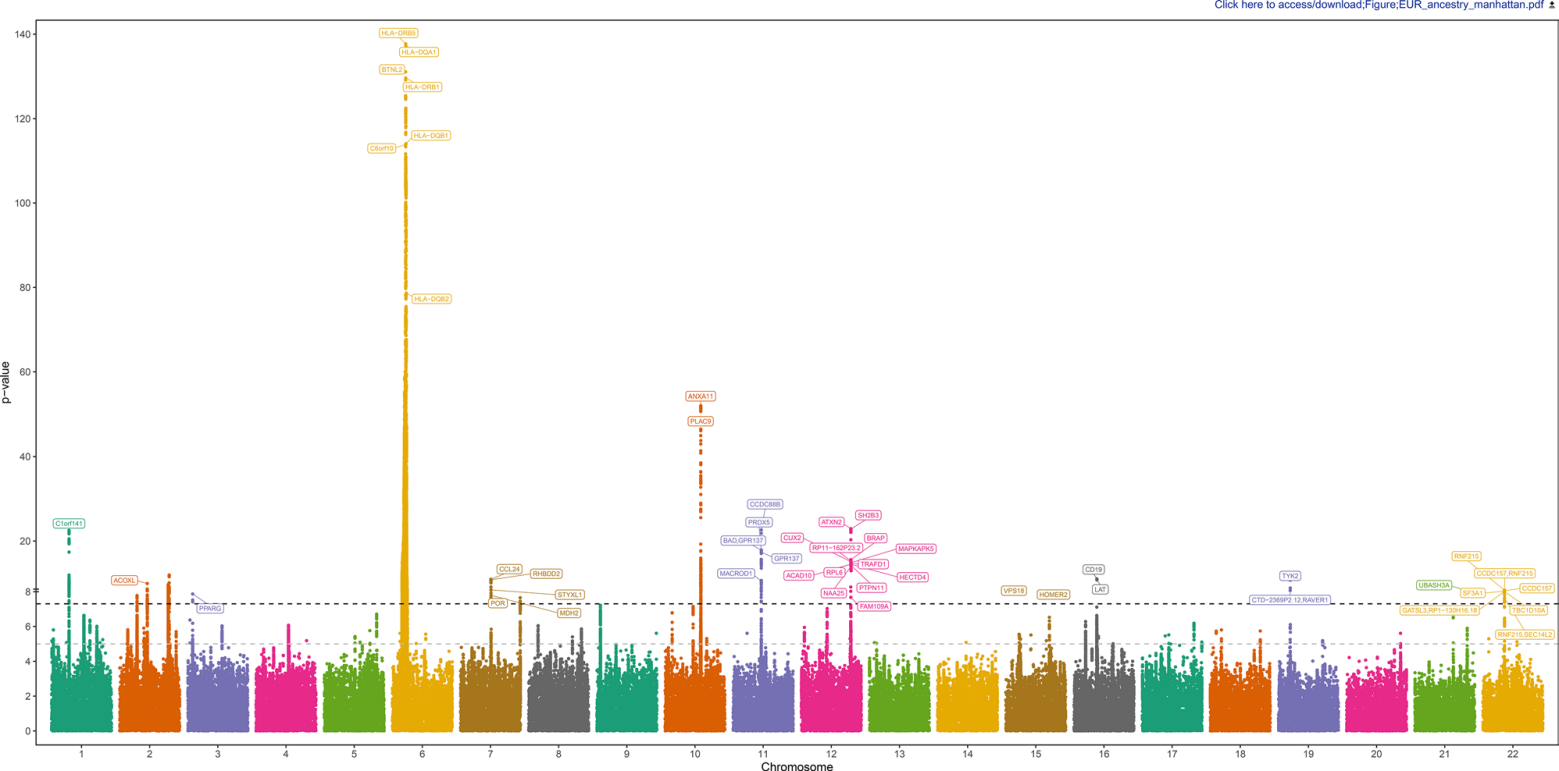
Manhattan plot for EUR ancestry meta-analysis

**Fig. 2:**
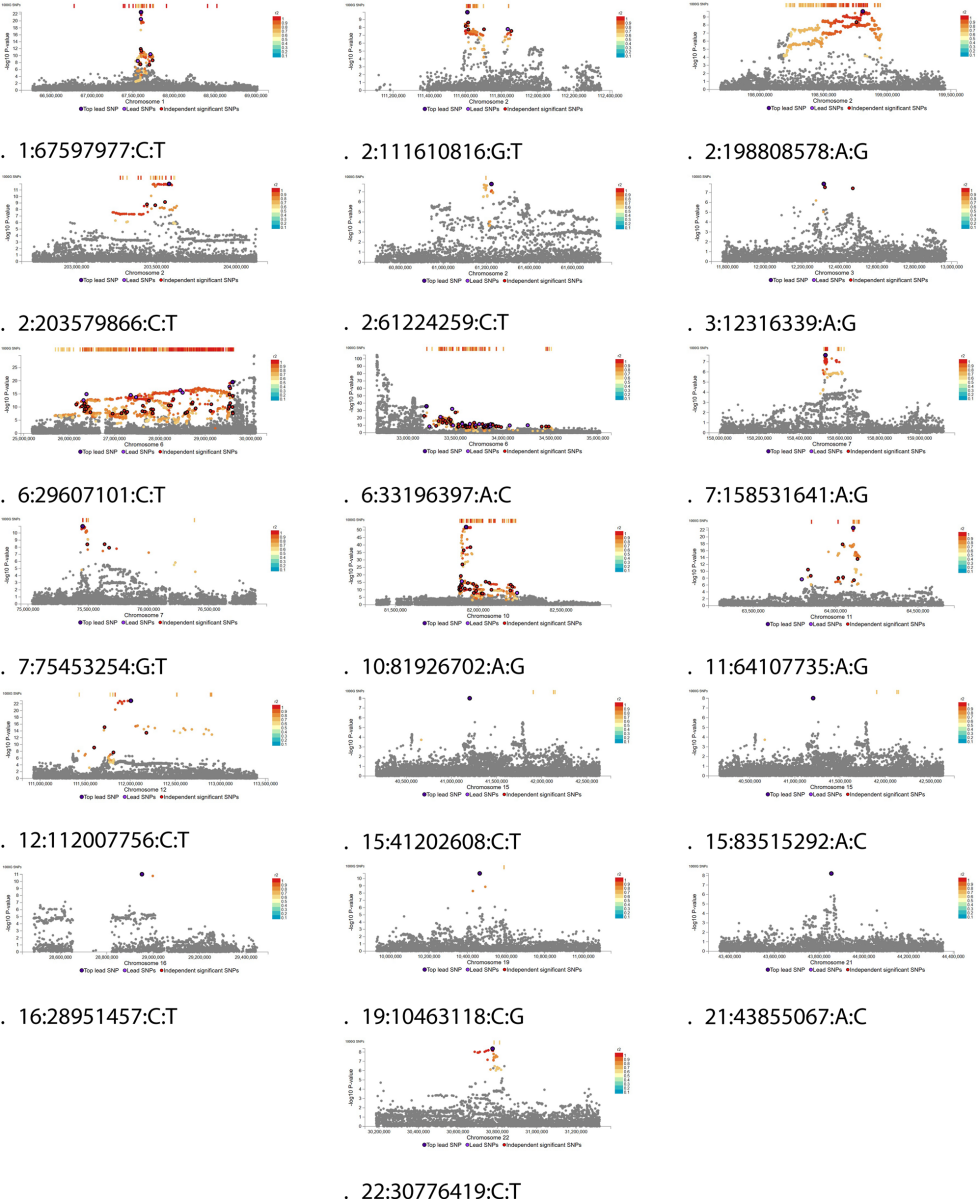
Regional plots from EUR ancestry meta-analysis

Nineteen risk loci were identified in EUR ancestry meta-analysis (Fig. 1, regional plots are reported in Fig. 2): of these, at least six (see Supplementary Table 1) had not been previously associated with the phenotype (at p-value < 5 × 10^−8^ significance level) in any single GWAS.

**Fig. 3:**
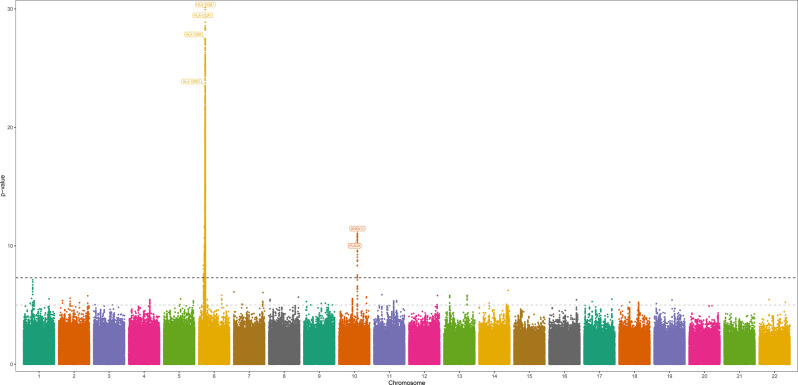
Manhattan plot for AFR ancestry meta-analysis

#### AFR ancestry

**Fig. 4:**
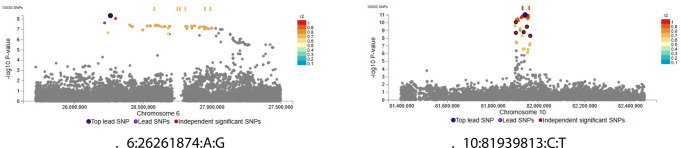
Regional plots from AFR ancestry meta-analysis

Two risk loci were identified in AFR ancestry meta-analysis (Fig. 3), mainly coinciding with results from the MVP summary statistics (Fig. 4 and Supplementary Files).

**Fig. 5:**
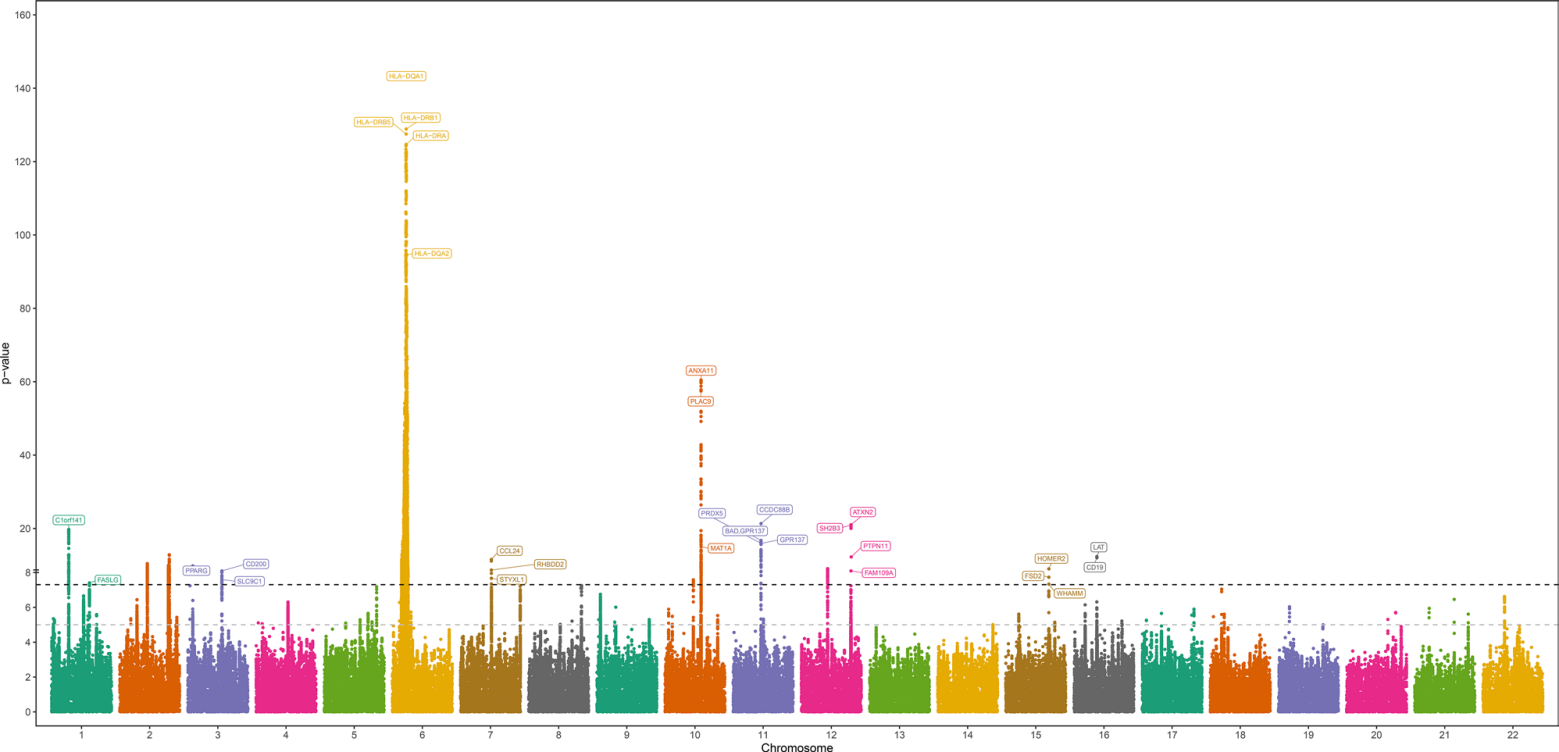
Manhattan plot for multi-ancestry meta-analysis

#### multi ancestry

**Fig. 6:**
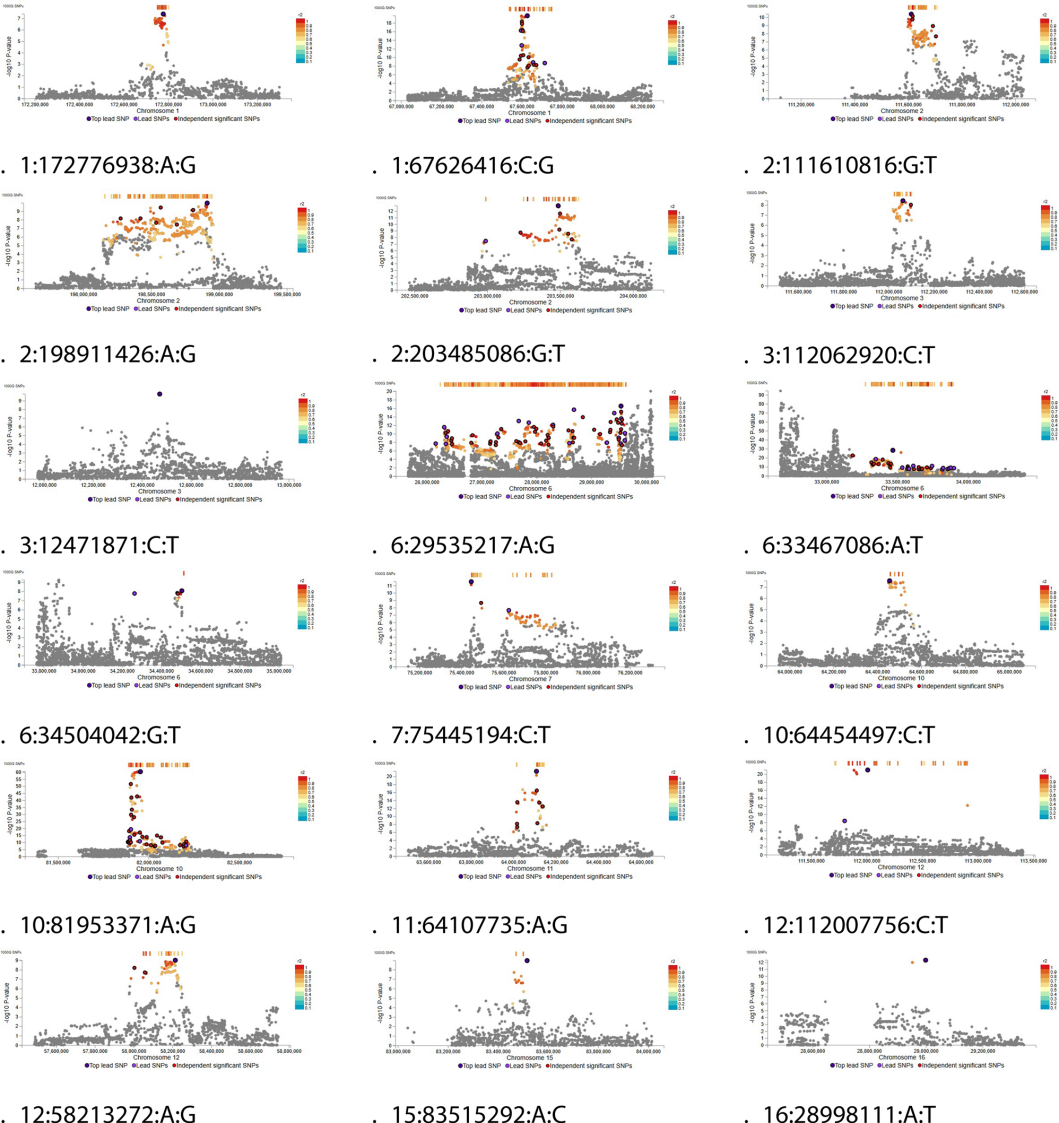
Regional plots from multi-ancestry meta-analysis

Eighteen risk loci were identified in multi-ancestry meta-analysis (Fig. 5): compared to EUR ancestry, additional loci were identified in chromosomes 1, 3, 10, and 12 (Fig. 6). Multi-ancestry meta-analysis was confronted with FinnGen+UKBB+MVP meta-analysis (released by FinnGen freeze 12) yielding consistent results.

### Genetic heritability

Total observed scale h^2^ in AFR ancestry was 0.1879 (0.2089), with *λ* = 1.0016 (mean *χ*^2^: 1.0062, intercept: 0.9864). Total observed scale h^2^ in EUR ancestry was 0.2504 (0.0581), with *λ* = 1.0436 (mean *χ*^2^: 1.1116, intercept: 0.9469). Overall, mild-moderate and moderate genetic heritability was estimated for AFR and EUR ancestry, respectively; mild residual genomic inflation could be observed in EUR ancestry.

### Functional annotation of variants

#### Tissue expression

**Fig. 7:**
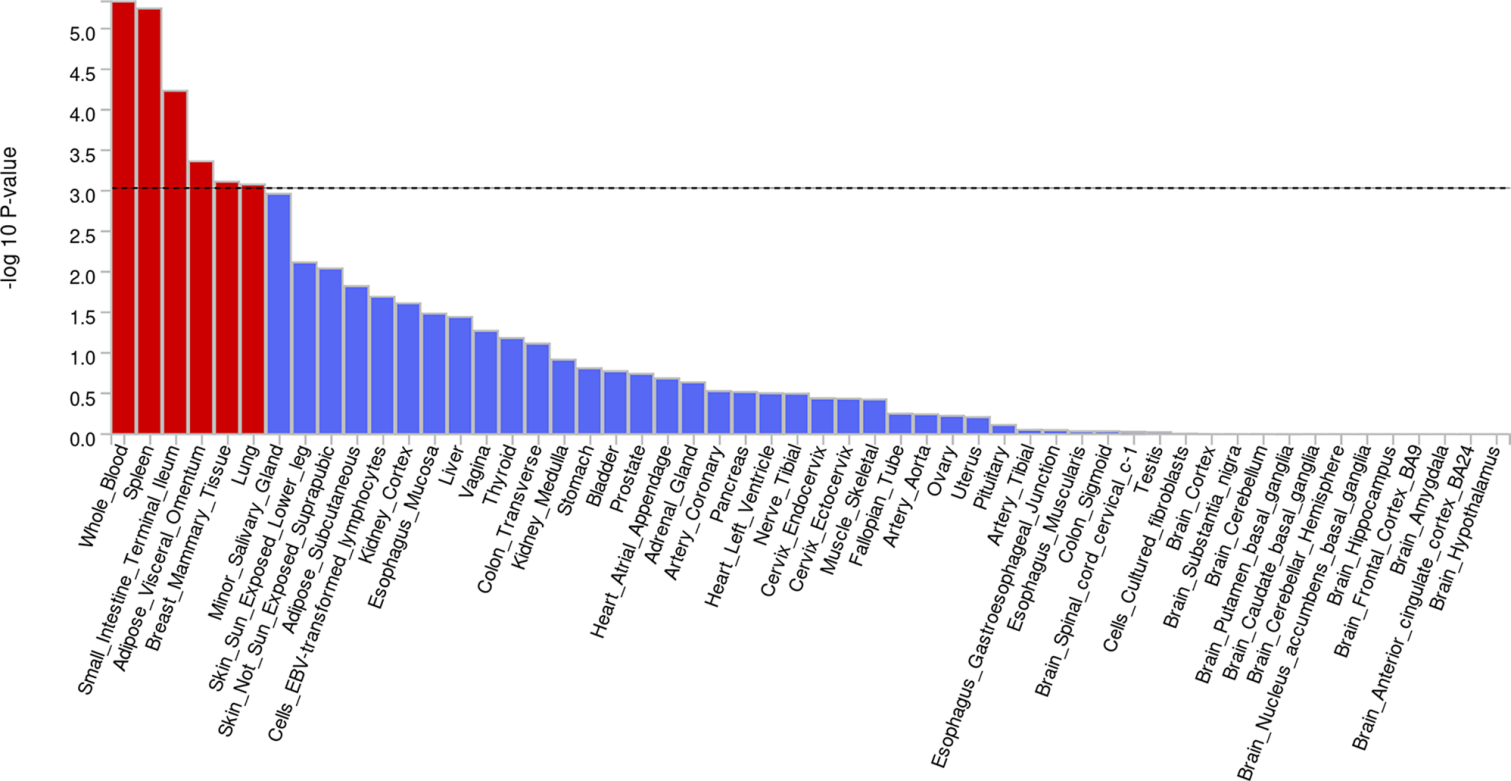
Tissue-enrichment analysis for EUR ancestry

The most significantly represented tissues (p-value < 10^−3^) in the EUR ancestry meta-analysis were whole blood, spleen, terminal ileum, visceral omentum, mammary tissue, and lung (Fig. 7). TEA in multi ancestry meta-analysis highlighted a significant association with whole blood and spleen (Supplementary Figure S1). No tissues in AFR meta-analysis were associated with the phenotype at the required significance level; however, the most significantly represented tissues were adrenal gland, kidney cortex, and pancreas (Supplementary Figure S2).

#### Assessment of significance genes and SNPs

Critical assessment of putative functional roles for non-MHC genes significantly associated with the phenotype in EUR and multi ancestry is reported in Table 1. Overall, SNPs from the following genes have already been identified in previous studies on sarcoidosis (GWAS or other): *CCDC88B*, *ANXA11*, *IL23R*, *FAM117B*, *CCL24*, *ATXN2/SH2B3*. The following additional genes have been associated with sarcoidosis through functional or other experimental studies: *PLCL1*, *PPARG*, *CD19*. Indirect evidence of some involvement in the pathogenesis of sarcoidosis is available for *PUS10* and *ESYT2*. The following additional genes have been involved in immune (dys)regulation: *RNF215*, *RPS6KA4*, *LAT*, *UBASH3A*, *HOMER2*, *SUMO2P* (indirect evidence). Finally, no clear evidence linking *ACOXL*, *MAT1A*, or *MACROD1* to sarcoidosis or immune (dys)regulation was found from the available literature.

**Table 1:** Genes significantly associated with the phenotype were critically reviewed on the basis of known mechanisms of action, available evidence of a role in sarcoidosis or related phenotypes, and previous identification in GWAS studies (rsids which have been identified both in the literature and in the present study are in bold). Comparison with the very recent study by Yuan et al. [28] could not be performed as complete summary statistic could not be accessed yet

Gene/Protein	Mechanism	Evidence	GWAS
*CCDC88B*/GIPIE	• role in suppressing apoptosis induced by endoplasmic reticulum stress in endothelial cells ( [29], not replicated in [30]) • contributes to the maturation and activation of T cells [30]	• expressed in lung cells [31]; has a role in decreasing apoptosis by binding GRP78 and IRE1, thus attenuating the IRE1/ASK1/JNK signaling pathway and expression of CHOP [29] • expressed in CD3+ cells (lymph nodes, thymus and spleen); its absence impairs T cell function and leads to reduced cytokine production [30]	Identified in [31]: • **rs6633743** (high regulatory potential) • rs647152 (possible influence on protein function) • rs671976 (probable causative variant or in LD with causative SNPs)
RNF215	• negatively regulates type 1 IFN [32]	• increased RNF215 expression blocksNF-*κ*B p65 binding to the IFNB1 promoter [32], thus preventing IFN*β* transcription [33]	-
ANXA11	• regulates apoptosis in immune cells [34]	• downregulated in immune cells exposed to activation stimuli [35]	Identified in [35, 36]: • **rs1049550** (C allele is associated with sarcoidosis risk) and [36]; • rs2784773 (strongly associated with sarcoidosis) [35])
IL23R	• part of the IL23/IL17 signaling pathway leading to the production of various cytokines [37] and involved in granuloma formation [38] • encodes for a subunit of the IL23 receptor [6], which consists of IL12R*β*1 and IL23R and is found on CD4+ memory T cells [39] • differentiation in Th17/Th IL-17 cells and consequent production of cytokines such as IL17 and TNF*α* is attributed to the binding of IL23 to its receptor on CD4+ naive T cells [37] • IL23R stimulates the production of IFN*γ* by CD45RO memory T cells [39]	• Th17/Th IL-17 cells are increased in peripheral blood and in the broncho-alveolar lavage (BAL) and are found within the granulomas of patients with sarcoidosis [40] • most sarcoidosis-related uveitis are genetically susceptible to IL23R [41]; • no efficacy was demonstrated for ustekinumab in pulmonary sarcoidosis compared to placebo [42]; • IL23 is upregulated in cutaneous sarcoidosis [43]	• rs11465804 and rs11209206 are negatively associated with sarcoid-related uveitis [44]; • the frequency of **rs11209026** is decreased in patients with sarcoidosis and in patients with sarcoidosis and uveitis [6] • rs117633859 and rs6664119 are associated with reduced serum expression of IL23R [6]; • rs12069782 in the IL23R promoter was associated with sarcoidosis in [31] • rs117633859 and **rs117282985**, upstream IL23R, are significantly associated with sarcoidosis in Japanese population [6]
FAM117B	• role in oxidative stress by activating KEAP1/NRF2 signaling pathway [45, 46]	• associated with sarcoidosis in [47]	**rs6748088** is significantly associated with susceptibility for sarcoidosis in EUR [31]
ACOXL	• role in oxidative stress (poor evidence) [48] • implicated in fatty acids metabolism [48]	-	-
PLCL1	• involved in NLRP3 activation [49]	• correlated to sarcoidosis in [50] • regulates fibroblasts through activation of NLRP3 inflammatory pathway in rheumatoid arthritis [49]; NLRP3 has been linked to Th17/Treg activation (with Th17 being a key player in the inflammation in sarcoidosis) [51] • the blockade of NLRP3 modifies Th17/Treg balance; • [50] directly correlates NLRP3 to sarcoidosis in granuloma formation	-
PPARG	• involved IFN*γ* production and Th17 activity [52, 53]	• in PPAR*γ* knock-out mice, the instillation of MWCNT allowed the creation of a murine model of sarcoidosis [54, 55] • reduced in alveolar macrophages of patients with pulmonary sarcoidosis [52] • activation of PPAR*γ* inhibits granuloma formation and reduces levels of inflammatory mediators CCL2 and osteopontin by decreasing NF-*κ*B [56]	-
CCL24	• involved in Th2 inflammatory response [6] and in pulmonary fibrosis [57]	• reduction in CCL24 expression may reduce Th2 inflammatory response favoring Th1 inflammatory response [6] • in systemic sclerosis, the blockade of CCL24 leads to a reduction in lung inflammation and fibrosis [87] • CCL24 is incresed in the BAL of sarcoidosis stage 3 patients compared to patients with Lo¨fgren syndrome [58] and in the acqeous humor of patients with uveits [59]	Significantly associated with sarcoidosis in [6] the risk allele rs4728493 (intronic) is associated with a reduced expression of CCL24 and in particular to Lo¨fgren syndrome
MAT1A	• putative role in oxidative stress [60]	-	-
RPS6KA4/MSK2	• regulation of inflammation [61]	• reduced levels of MSK1 and MSK2 are correlated with an increase in TNF, IL-6 and IL-12 [61, 62]	-
MACROD1	• involved in inflammation [63]	-	-
ATXN2,SH2B3	-	-	Identified in [31]: • **rs3184504** • **rs653178** significantly associated with Lo¨fgren Syndrome
LAT,RP11- 231C14.10, RP11-264B17.3	• linker for activation of T cells [64]	• T cell aberrant activation has been implicated in disease outcome [65] • the role of T cells in the pathogenesis of sarcoidosis has been extensively studied [66]	-
PUS10	• plays a direct role in apoptotic pathways through a positive feedback mechanism involving caspase-3 • defects in PUS10 transport or interactions lead to increased cell survival [67]	• BAL fluid lymphocytes from patients with sarcoidosis display a non-apoptotic morphology associated with endogenous caspase-3 activity [68]	-
ESYT2	• lipid transport protein involved in plasma membrane lipid homeostasis [69] that clears diacylglycerol from the plasma membrane leading to down regulation of T-cells activation and reduction of IL-2 levels [70] • involved in fibroblasts migration (art 4)	• absence of ESTY2 leads to high levels of diacylglycerol in the plasma membrane, thus increasing the activation of T cells • increases TCR signaling, stimulates CD4+ production of IL2 [70], a Th1-like cytokine whose expression in CD4+ and CD8+ T cells is increased in the BAL fluid of patients with sarcoidosis compared to controls [71] • mice with ESYT2 gene deficiency shows an altered embryogenic fibroblasts migration [72]	-
UBASH3A/STS2	• involved in reducing the activation and signaling of T cells and in the expression of cytokines such as IL-2 and IFN*γ* [73] • inhibits NF-*κ*B signaling in type 1 diabetes [74]	• STS-2 and STS-1 knock-out mice show an augmented response following T cell receptor (TCR) stimulation, enhanced levels IFN*γ* and IL2 compared to wild type mice [73] • knock-out for STS2 alone has a weaker role in TCR stimulation and IFN*γ* production [75] • ZAP70 is a transducer for TCR [76] and defect of UBASH3A/STS-2 and UBASH3B/STS-1 lead to enhanced ZAP70 activation through phosphorylation [73, 76] while defect in STS-2 alone is associated with weak ZAP70 phosphorylation [75] • overexpression of UBASH3A reduces IL2 and NF-*κ*B levels [74]	-
HOMER2	• acts as a negative regulator of T cell activation by modulating TCR and CD28 signaling • plays a role in regulating NF-*κ*B, NFAT, and calcium signaling [77]	• following stimulation with anti-CD3, T cells from Homer triple knock-out mice produced IL-2 in amounts 2 to 6 times higher compared to wild type.	-
SUMO2P	• alterations in SUMOylation levels influence the differentiation of regulatory T cells (Tregs) and the transcription of IL-17 in Th17 lymphocytes [78]	• although there is no direct evidence that alterations in the SUMO-2P1 pseudogene are associated with immune system dysregulation, SUMOylation itself has been implicated in immune regulation	-
CD19	• B cells and Tfh2- and Tfh17-like cells – most effective cell type in supporting B-cell activity, particularly antibody production – may play a role in the occurrence and development of sarcoidosis and other autoimmune conditions [79]	• significant lymphopenia involving CD4, CD8, and CD19 positive cells was common in sarcoidosis patients and correlated with disease severity [80]	-

For each risk locus in EUR-ancestry meta-analysis, SNPs with CADD score > 12.37 were manually selected and reported in Table 2. In AFR ancestry meta-analysis, only two SNPs displayed CADD score. >12.37 (see Supplementary files).

**Table 2:** For each risk locus in EUR ancestry meta-analysis, mapped SNPs with CADD score *>*12.37 and r^2^*>*0.6 are reported under the lead variant (first row in bold)

risk locus	rsid	bp	_**r**_**2**	GWAS P-value	Annotation	Nearest Gene	CADD
**1:67597977:C:T**	**rs2024825**	**67597977**	**1**	2.96E-23	**intronic**	**C1orf141**	**3.432**
	rs11209026	67705958	0.920359	1.268E-10	exonic	IL23R	26.5
	rs11208997	67560956	0.642851	0.00226	exonic	C1orf141	12.62
	rs2224501	67532526	0.619157	0.000000004463	intergenic	SLC35D1	13.75
	rs4655514	67550921	0.650973	0.001956	intergenic	C1orf141	15.59
	rs7519768	67555522	0.629107	5.933E-10	intergenic	C1orf141	12.99
	rs34388889	67563430	0.774939	NaN	intronic	C1orf141	12.53
	rs12140736	67586671	0.631274	1.427E-10	intronic	C1orf141	13.86
**2:61224259:C:T**	**rs10181042**	**61224259**	**1**	1.67E-08	**intronic**	**PUS10**	**0.43**
	rs6710043	61218280	0.787821	0.00009042	intronic	PUS10	13.93
**2:111610816:G:T**	**rs10183338**	**111610816**	**1**	1.16E-10	**intronic**	**ACOXL**	**5.723**
	rs1554005	111598958	1	3.15E-08	exonic	ACOXL	17.28
	rs6732565	111607832	0.89891	5.95E-09	intronic	ACOXL	15.15
	rs13401811	111616104	1	1.83E-08	intronic	ACOXL	21.5
	rs17525147	111619522	0.919722	4.95E-08	intronic	ACOXL	16.43
	rs9308690	111638898	0.828115	3.69E-08	intronic	ACOXL	12.69
	rs9308692	111641263	0.828115	3.57E-08	intronic	ACOXL	13.03
	rs28811027	111653937	0.834575	3.11E-08	intronic	ACOXL	12.39
	rs113122861	111657687	0.858706	NaN	intronic	ACOXL	14.45
	rs1513826	111660710	0.834575	4.00E-08	intronic	ACOXL	13.45
**2:198808578:A:G**	**rs1401090**	**198808578**	**1**	1.98E-10	**intronic**	**PLCL1**	**0.618**
	rs788018	198265526	0.739653	0.000002647	exonic	SF3B1	18.96
	rs788023	198283305	0.739653	0.000002588	exonic	SF3B1	13.29
	rs8539	198362018	0.740639	0.000001763	exonic	HSPD1	18.44
	rs1064213	198950240	0.773667	0.0000001944	exonic	PLCL1	26.7
	rs788007	198233676	0.710917	0.00000405	intergenic	NPM1P46	16.84
	rs67657812	198387401	0.698702	0.00000002455	intronic	HSPE1–MOB4:MOB4	12.46
	rs3838584	198416728	0.74175	NaN	UTR3	MOB4	15.71
	rs11336956	198540701	0.901653	NaN	UTR5	RFTN2	13.6
	rs700662	198668751	0.944576	0.00000001692	upstream	PLCL1	12.6
	rs696817	198722225	0.954087	0.000000008587	intronic	PLCL1	13.92
	rs570036157	198723904	0.954087	NaN	intronic	PLCL1	12.66
	rs4850812	198743655	0.953646	0.000000008505	intronic	PLCL1	15.42
	rs938929	198780860	0.981324	0.000000001489	intronic	PLCL1	12.86
	rs1464211	198798630	0.944517	0.00000001046	intronic	PLCL1	12.99
	rs55696134	198821569	0.981393	4.065E-10	intronic	PLCL1	12.56
	rs61435657	198825506	0.627221	0.00006861	intronic	PLCL1	14.38
	rs5837576	198826363	0.94	NaN	intronic	PLCL1	15.33
	rs13382697	198920560	0.81266	0.0000003302	intronic	PLCL1	15.25
**2:203579866:C:T**	**rs191390916**	**203579866**	**1**	1.16E-12	**intronic**	**FAM117B**	**1.08**
	rs72925089	203359566	0.961832	5.25E-08	intronic	BMPR2	17.28
	rs140974562	203460202	0.713015	NaN	intergenic	AC009960.1	15.01
	rs933969	203499720	0.646164	NaN	upstream	FAM117B	13.85
	rs6748088	203556526	0.973392	1.39E-12	intronic	FAM117B	13.85
**3:12316339:A:G**	**rs2972166**	**12316339**	**1**	1.36E-08	**intergenic**	**PPARG**	**0.377**
**6:29607101:C:T**	**rs3131856**	**29607101**	**1**	3.20E-20	**intergenic**	**SUMO2P1**	**4481**
	rs13195402	26463575	0.877433	1.17E-13	exonic	BTN2A1	23.7
	rs13195509	26463660	0.755787	2.664E-13	exonic	BTN2A1	22.5
	rs139332558	26637724	0.889731	NaN	exonic	ZNF322	19.82
	rs7756481	27115069	0.879068	0.00000139	exonic	HIST1H2AH	16.39
	rs141138864	27223064	0.704895	NaN	exonic	PRSS16	15.96
	rs16897515	27278020	0.966338	1.82E-08	exonic	POM121L2	19.9
	rs200484	27775674	0.809201	7.57E-15	exonic	HIST1H2BL	16.23
	rs200981	27833174	0.782008	7.32E-15	exonic	HIST1H2AL	15.9
	rs200956	27839746	0.949039	3.42E-09	exonic	HIST1H3I	16.41
	rs200973	27858421	0.984932	1.88E-09	exonic	HIST1H3J	21.5
	rs34788973	27879200	0.947228	4.47E-16	exonic	OR2B2	23.2
	rs61742093	27879982	0.947228	9.87E-16	exonic	OR2B2	22.6
	rs1679709	28228342	0.844255	9.92E-11	exonic	NKAPL	22.3
	rs33932084	28268824	0.940768	8.94E-14	exonic	PGBD1	14.75
	rs2230683	28891176	0.92562	1.21E-17	exonic	TRIM27	18.69
	rs404240	29523957	0.897948	1.45E-15	exonic	UBD:GABBR1	14.02
	rs6902389	26322153	0.729201	1.05E-11	ncRNA exonic	HIST1H3PS1	13.92
	rs6908156	26322861	0.729201	1.05E-11	upstream	HIST1H3PS1	14.27
	rs6902392	26322115	0.729201	1.05E-11	ncRNA exonic	HIST1H3PS1	14.9
	rs9467740	26383250	0.872107	7.73E-12	upstream	BTN2A2	12.85
	rs3832422	26383523	0.872107	NaN	UTR5	BTN2A2	13.39
	rs12207181	26431285	0.847182	7.81E-10	ncRNA exonic	BTN2A3P	12.45
	rs34104395	26478252	0.755787	2.82E-13	intergenic	BTN2A1	13.34
	rs13198716	26582035	0.890433	1.49E-13	intergenic	ABT1	12.54
	rs9393735	26582327	0.719829	4.74E-07	intergenic	ABT1	12.51
	rs13203358	26590578	0.674991	2.01E-07	intergenic	ABT1	13.55
	rs6456742	26617075	0.785548	2.24E-08	intergenic	RP11-457M11.6	12.79
	rs72845515	26646871	0.785548	2.02E-08	intronic	ZNF322	12.63
	rs12198053	26667659	0.664209	5.50E-08	intergenic	ZNF322	13.55
	rs12192446	26995638	0.896444	3.23e-7	intergenic	LINC00240	13.3
	rs72838249	26998489	0.925126	9.65E-08	intergenic	VN1R12P	13.41
	rs72838262	27008517	0.925126	1.58E-07	intergenic	VN1R12P	13.7
	rs6934329	27158033	1	2.40E-08	intergenic	RP11-209A2.1	13.14
	rs12198077	27169307	0.868364	0.000001443	intergenic	RP11-209A2.1	12.54
	rs72843629	27175215	0.868364	9.54E-07	intergenic	RP11-209A2.1	12.52
	rs67457459	27198343	0.711219	1.45E-10	intergenic	PRSS16	13.45
	rs72843641	27203335	0.858902	8.25E-07	intergenic	PRSS16	15.71
	rs72839477	27327000	1	8.09E-15	ncRNA exonic	ZNF204P	14.36
	rs34573979	27480526	0.986328	2.38E-13	intergenic	XXbac-BPGBPG34I8.1	15.35
	rs56405707	27640246	0.986328	1.08E-13	intergenic	RP1-15D7.1	13.34
	rs9357045	27688927	0.690251	3.36E-07	intergenic	RP1-97D16.1	19.07
	rs9348774	27688930	0.690251	4.66E-07	intergenic	RP1-97D16.1	17.07
	rs13193480	27702561	0.972983	6.80E-14	intergenic	RP1-97D16.1	15.29
	rs4713119	27712825	0.955161	8.27E-09	intergenic	RP1-97D16.1	12.66
	rs9468225	27745719	0.609622	0.000007862	intergenic	RSL24D1P1	17.65
	rs200483	27774824	0.809201	5.99e-15	upstream	HIST1H4PS1	13.69
	rs17751184	27775028	0.972983	4.02E-14	ncRNA exonic	HIST1H4PS1	14.92
	rs375576927	27835322	1	NaN	UTR5	HIST1H1B	13.52
	rs200949	27835435	0.737187	2.81E-14	upstream	HIST1H1B	12.63
	rs45509595	27840926	0.972983	1.74E-15	UTR3	HIST1H4L	15.55
	rs184666393	27870324	1	NaN	intergenic	RNU7-26P	17.84
	rs71559054	27896799	0.947228	5.69E-16	intergenic	OR2W6P	14.09
	rs67040724	27905509	0.947228	2.84E-16	ncRNA exonic	OR2W6P	13.54
	rs148418547	27914359	0.947228	NaN	intergenic	OR2W6P	13.54
	rs28360499	27945396	0.934796	5.44E-16	ncRNA exonic	OR2W4P	13.56
	rs149947	27972433	0.692362	0.000009017	intergenic	IQCB2P	13.59
	rs149900	28014597	0.708989	0.00001664	ncRNA exonic	OR2B7P	14.67
	rs35572414	28015111	0.750238	NaN	ncRNAexonic	OR2B7P	12.49
	rs203888	28021589	0.683285	0.000008666	ncRNA exonic	OR2B8P	15.75
	rs35155115	28021853	0.774358	0.000006234	ncRNA exonic	OR2B8P	14.64
	rs188105	28071393	0.766448	0.000002204	intergenic	ZSCAN12P1	15.23
	rs75874576	28087832	0.902652	2.66E-12	intergenic	ZSCAN16–AS1	12.49
	rs9468300	28126840	0.66646	2.69E-10	UTR3	ZKSCAN8	13.42
	rs1736904	28219270	0.837331	2.04E-08	intronic	ZKSCAN4	12.41
	rs146407472	28243175	1	NaN	ncRNA intronic	RP5-874C20.3	14.64
	rs13211507	28257377	0.955051	1.58E-16	intronic	PGBD1	13.79
	rs36005309	28277346	0.955051	NaN	intergenic	PGBD1	12.91
	rs56075693	28290328	0.969715	1.24E-16	intergenic	ZSCAN31	12.71
	rs10591593	28315542	0.609775	NaN	intronic	ZSCAN31	12.96
	rs35744819	28318331	1	1.01E-16	intronic	ZSCAN31:ZKSCAN3	13.57
	rs78371185	28321502	1	NaN	intronic	ZSCAN31:ZKSCAN3	14.17
	rs71559081	28372069	0.660174	0.00004601	intergenic	ZSCAN12	12.97
	rs13209596	28396190	0.660174	0.00004634	intergenic	ZSCAN23	13.61
	rs7765989	28400295	0.703367	0.000005804	intronic	ZSCAN23	12.95
	rs11967609	28656109	0.780705	3.49E-07	intergenic	LINC00533	13.29
	rs12193659	28677144	0.899801	4.65E-12	intergenic	RPSAP2	14.4
	rs1233583	28715566	0.926851	4.83E-17	intergenic	RPSAP2	14.21
	rs9257189	28757555	0.926851	2.93E-17	intergenic	NOL5BP	14.92
	rs209181	28792477	1	3.66E-10	intergenic	XXbac-BPG308K3.5	12.38
	rs3135300	28824397	0.911716	1.64e-17	intergenic	XXbac-BPG308K3.6	13.51
	rs3135302	28829637	0.911716	1.81E-17	ncRNA exonic	XXbac-BPG308K3.6:RPL13P	12.44
	rs3132392	28838629	0.911716	2.25e-17	intergenic	XXbac-BPG308K3.6	13.32
	rs3130746	29153155	0.92562	5.32E-17	intergenic	OR2J4P	12.86
	rs3129093	29170261	1	4.64E-09	intergenic	OR2H4P	13.4
	rs426595	29512482	0.865438	6.44E-08	intergenic	GPR53P	14.95
	rs111508444	29603512	1	1.03E-10	downstream	SUMO2P1	19
**6:33196397:A:C**	**rs34859217**	**33196397**	**1**	1.71E-36	**intergenic**	**HTATSF1P**	**3.039**
	rs9394145	33399778	0.881875	4.646E-13	exonic	SYNGAP1	17.25
	rs4713668	33690796	0.945819	1.426E-11	exonic	IP6K3	22.1
	rs114699053	33172306	1	1.38E-23	upstream	SLC39A7	13.33
	rs211448	33343859	1	4.33E-22	intergenic	LYPLA2P1	12.78
	rs116323349	33401291	0.660355	1.39e-16	intronic	SYNGAP1	16.5
	rs9366821	33444074	0.776956	8.45E-20	intergenic	ZBTB9	14.14
	rs62405954	33524820	0.874942	5.64E-29	intergenic	BAK1	12.68
	rs210122	33574635	0.980196	3.85e-12	intergenic	ITPR3	12.96
	rs9368768	33588610	0.752623	0.0000002741	UTR5	ITPR3	15.94
	rs71565395	33593577	0.904311	1.07e-9	intronic	ITPR3	13.36
	rs749847	33665001	0.932443	2.332E-11	UTR3	UQCC2	13.46
	rs9469566	33667605	0.949405	1.76e-11	intronic	UQCC2	14.21
	rs4713659	33667839	0.949405	1.948E-11	intronic	UQCC2	14.48
	rs6457739	33673831	0.949405	1.937E-11	intronic	UQCC2	20.2
	rs58420296	33677214	0.932443	1.103E-11	intronic	UQCC2	13.5
	rs2966	33689520	0.925707	1.972E-11	UTR3	IP6K3	14.81
	rs2967	33689534	0.662091	0.004241	UTR3	IP6K3	12.69
	rs4713674	33708169	0.844356	3.958E-12	intronic	IP6K3	16.92
	rs73747318	33782048	0.796913	0.0000002631	intergenic	MLN	12.44
	rs17600945	33802263	0.88096	0.000005024	intergenic	MLN	20.9
	rs7746977	33842481	0.67781	2.555E-10	ncRNA intronic	LINC01016	14.81
	rs11758326	34482498	0.809188	0.00000002457	intronic	PACSIN1	18.95
**7:75453254:G:T**	**rs7811626**	**75453254**	**1**	1.13E-11	**upstream**	**CCL24**	**1.552**
**7:158531641:A:G**	**rs7803766**	**158531641**	**1**	2.25E-08	**intronic**	**ESYT2**	**1.718**
	rs2305473	158536267	0.992399	0.00000005877	exonic	ESYT2	22.2
	rs2305475	158536345	0.992399	0.0000000623	exonic	ESYT2	22.7
	rs1061735	158526455	0.947305	0.000001459	UTR3	ESYT2	16.15
	rs842450	158567423	0.687358	0.000001523	intronic	ESYT2	14.26
	rs842452	158575440	0.687358	0.000001293	intronic	ESYT2	12.61
**10:81926702:A:G**	**rs1049550**	**81926702**	**1**	9.08E-53	**exonic**	**ANXA11**	**28.6**
	rs12411782	82012967	0.70449	0.000003082	exonic	AL359195.1	18.65
	rs2573360	81891731	1	4.134E-10	intronic	PLAC9	14.58
	rs2819885	81892002	0.65768	1.154E-11	intronic	PLAC9	12.69
	rs2819875	81903517	0.782327	4.26E-39	intronic	PLAC9	12.57
	rs3851053	81918582	0.635924	4.48E-28	intronic	ANXA11	14.57
	rs141872729	81927935	0.979479	NaN	intronic	ANXA11	15.34
	rs11202248	81991475	0.930316	3.77E-11	intergenic	RP11-40F6.1	13.82
	rs60492867	82022560	0.88442	NaN	intergenic	MAT1A	14.14
	rs10788579	82108610	0.962307	2.57E-14	intronic	DYDC1:DYDC2	13.51
	rs35050604	82196677	0.956171	4.65E-14	ncRNA exonic	RP11-137H2.6	12.39
	rs10887881	82204620	0.987203	3.29E-10	intergenic	RP11-137H2.6	15.41
	rs7095954	82209232	0.84176	5.37E-08	intergenic	TSPAN14	12.88
	rs116857892	82214065	0.84176	4.91E-08	UTR5	TSPAN14	13.16
	rs12220642	82218964	0.707937	0.000001754	intronic	TSPAN14	12.7
**11:64107735:A:G**	**rs663743**	**64107735**	**1**	2.38E-23	**UTR5**	**CCDC88B**	**5934**
	rs647152	64109118	0.807175	1.64E-18	exonic	CCDC88B	13.15
	rs600377	64124940	0.682994	5.377E-11	exonic	CCDC88B	14.93
	rs521950	64127744	0.682994	3.66E-11	exonic	RPS6KA4	14.41
	rs11542299	64138805	0.876014	4.45E-15	exonic	RPS6KA4	16.99
	rs60031276	64012910	0.747794	2.52E-15	UTR5	PPP1R14B	18.33
	rs35989122	64011614	0.992329	NaN	downstream	FKBP2	14.87
	rs11231727	64011854	0.90993	0.0000001063	downstream	PPP1R14B	12.39
	rs2510066	64052447	0.80836	4.22E-18	UTR5	GPR137	13.7
	rs542907	64124980	0.682994	1.097E-10	UTR3	CCDC88B	16.01
**12:112007756:C:T**	**rs653178**	**112007756**	**1**	1.20E-23	**intronic**	**ATXN2**	**0.312**
	rs7968960	111426615	0.79915	8.10E-09	intergenic	RP1-46F2.2	15.51
	rs10849925	111495518	0.817697	0.0000001583	intronic	CUX2	16.4
	rs4378452	111504033	0.825654	0.00000006634	intronic	CUX2	13.24
	rs7399113	111792848	0.62465	0.00001513	intergenic	CUX2	17.49
**15:41202608:C:T**	**rs181956065**	**41202608**	**1**	9.59E-09	**intergenic**	**RP11-540O11.1**	**15.11**
**15:83515292:A:C**	**rs11856316**	**83515292**	**1**	1.32E-08	**UTR3**	**HOMER2**	**2.074**
**16:28951457:C:T**	**rs11645302**	**28951457**	**1**	9.45E-12	**downstream**	**CD19**	**1.707**
**19:10463118:C:G**	**rs34536443**	**10463118**	**1**	1.95E-11	**exonic**	**TYK2**	**25.5**
	rs74956615	10427721	0.828325	5.24E-09	UTR3	CTD-2369P2.12:RAVER1	20.8
**21:43855067:A:C**	**rs1893592**	**43855067**	**1**	6.01E-09	**intronic**	**UBASH3A**	**11.16**
**22:30776419:C:T**	**rs757870**	**30776419**	**1**	4.34E-09	**intronic**	**RNF215**	**0.304**
	rs757660	30793137	0.800351	2.38E-08	exonic	RNF215:SEC14L2	19.14
	rs1860217	30685560	0.975458	9.45E-09	UTR5	GATSL3:RP1-130H16.18	15.03
	rs2240422	30700924	0.980324	1.15E-08	intronic	TBC1D10A	13.35

#### Gene-set enrichment analysis

**Fig. 8:**
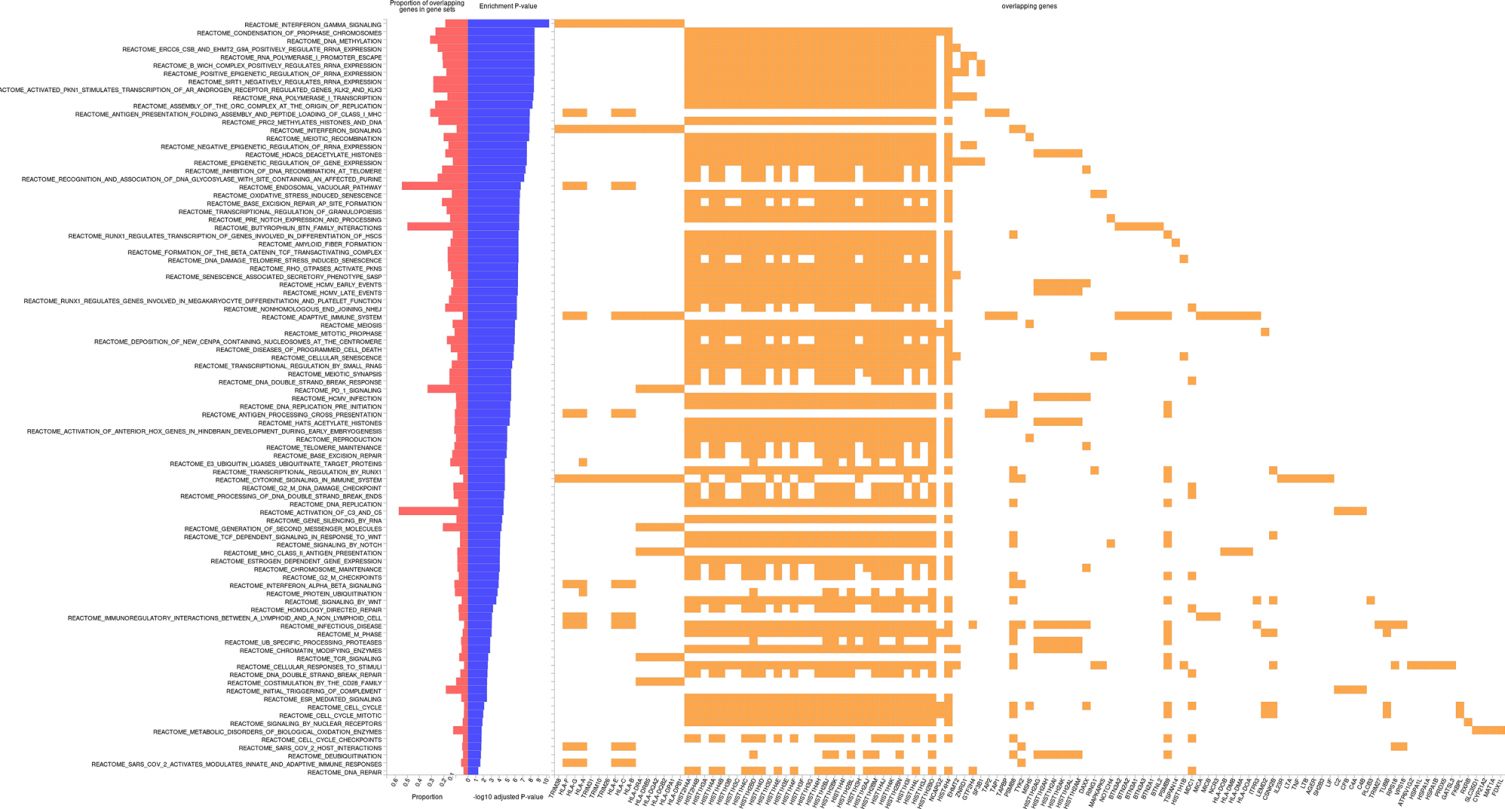
Gene-set enrichment analysis for EUR ancestry

Interferon gamma signaling, meiotic recombination/condensation of prophase chromosomes, and DNA methylation were the most enriched gene sets in EUR (Fig. 8) and multi-ancestry meta-analysis (Supplementary Figure S3). PD1 and INF*γ* signaling, generation of second messenger molecules, and MHC class II antigen presentation were the most enriched gene sets in AFR ancestry (Supplementary Figure S4).

## Discussion

In this study, we present a GWAS meta-analysis for sarcoidosis in European and African ancestries, aggregating publicly available summary statistics from the most recent releases of some of the largest genome biobanks. Moreover, our multi-ancestry meta-analysis is the first to include populations of African, European, and East Asian ancestries together.

To date, only few GWAS have selectively focused on sarcoidosis so far [6, 31]: Fisher et al. identified 4 risk loci, whereas Meguro et al. identified 3 risk loci. Two risk loci from both studies overlap with our findings (nearest genes: *ATXN2/SH2B3* and *FAM117B* in [31]; nearest genes: *CCL4* and *C1orf141-IL23R* in [6]). Very recently, Yuan et al. have presented a meta-analysis in which they studied in depth the same phenotype [28]. Our work, which was conducted independently, aims to add new evidence to their study, as we employed different techniques and accessed different summary statistics. For instance, we included the results from the BioBank Japan project and used the latest Finngen release, whose summary statistics are the most informative concerning this phenotype. Compared to Yuan et al., our analysis appears to be more restrictive, as we found less risk loci [28]. Our results, however, are comparable to the multi-ancestry meta-analysis issued by FinnGen and including patients from the MVP and UKBB [81].

In this regard, it is of interest that at least three risk loci (lead SNPs: rs10181042, rs7803766, rs181956065; nearest genes: *PUS10*, *ESYT2*, *RP11-540O11.1*) have been identified in our study but not reported elsewhere (see Supplementary file 1).

For functional annotation and subsequent analysis, European ancestry yielded the highest number of identifiable loci. In fact, the results from African ancestry meta-analysis mostly coincided with the single summary statistics from the MVP study, and only two known risk loci could be observed - although a potential trend was highlighted at least for a locus in chromosome 1, in the *IL23R* region. This is likely due to low sample numbers in controls, as well as - and especially - in cases of AFR ancestry.

This highlights the urgent need to extend efforts toward larger case-control populations in AFR (and EAS) ancestries, compared to European populations.

It is worthwile to note that multi-ancestry meta-analysis confirmed some statistically significant associations for trending loci in European ancestry, in particular in chromosomes 1 (lead SNP 1:67626416:C:G), 3 (lead SNP 3:12471871:C:T), 6 (lead SNP 6:34504042:G:T), 10 (lead SNP

10:64454497:C:T), and 12 (lead SNP 12:112007756:C:T). Meanwhile, some risk loci in chromosomes 7 (lead SNP 7:75453254:G:T), 15 (lead 15:41202608:C:T), 19 (lead SNP 19:10463118:C:G), 21 (lead SNP

21:43855067:A:C), and 22 (lead SNP 22:30776419:C:T) appeared private to EUR ancestry, as they lost statistical significance when including multiple ancestries (Figs. 6 and 4).

It is particularly interesting that tissue-enrichment analysis in European ancestry (Fig. 7) highlights a significantly enriched expression in tissues (whole blood, spleen) involved in immunitary functions and activity of the reticuloendothelial system. Additionally, TSEA shows enrichment in the lung, the primary site affected by the disease, as well as in other tissues—including the terminal ileum, visceral omentum, and mammary tissue—whose involvement in sarcoidosis remains less clear. Crucially, such findings are lost when performing multi-ancestry analysis (Figure S1), perhaps due to LD distortions caused by the coexistence of ancestry-specific r^2^ values.

In both European and African ancestries, mild-moderate genetic heritability of the trait has been evidenced, corroborating the assumption that sarcoidosis is a predominantly acquired autoimmune disease, to which some familiar risk appears to have an impact nonetheless.

Gene-set enrichment analysis in EUR and multi-ancestry meta-analysis yielded some particularly interesting results, as the most represented metabolic pathways in both analyses were related to interferon gamma signaling and DNA methylation.

Interferon-*γ* (IFN*γ*) signaling plays a pivotal role in driving the Th1-dominant immune response. Produced mainly by NK cells and Th1 lymphocytes, IFN-*γ* enhances macrophage activation and promotes the release of chemokines, facilitating the recruitment of Th1/17 cells, monocytes, Tregs, and B cells to inflamed lung tissue. It has been shown that this persistent immune activation fosters granuloma formation and sustains an exaggerated inflammatory response [82].

The hypothesis of methylation alterations as a risk factor for developing sarcoidosis is particularly intriguing, as this would adapt well to the aforementioned disease model in which both acquired and genetic risk factors coexist.

Growing evidence highlights the role of epigenetic regulation in sarcoidosis [83]. Transcriptional changes have been observed in peripheral blood cells [84, 85] and transbronchial biopsies [86], suggesting an epigenetic influence on disease mechanisms. DNA methylation and histone modifications play a role in the differentiation of CD4+ and regulatory T (Treg) cells [87, 88], potentially shaping immune responses. Additionally, genes involved in chromatin remodeling, such as *HDAC* and other chromatin-modifying factors, have been linked to the disease [86]. In this perspective, our findings could be seen as further evidence supporting a role for epigenetics in the pathogenesis of sarcoidosis. Be that as it may, caution must be exercised when interpreting these results, as the histone protein genetic region is widely overposed to the MHC.Therefore, over-representation due to some degree of LD cannot be ruled out (although, only in GSEA the MHC region was included in the analysis as an attempt to account for LD distortions).

Among the non-MHC genes that were represented in the risk loci (Table 1), at least 9 have been previously associated to sarcoidosis. In particular *CCDC88B*, *ANXA11*, *IL23R*, *FAM117B*, *CCL24*, and *ATXN2/SH2B3* have already been identified in previous GWAS or other genetic studies. Among those, *CCDC88B* and *ANXA11* are potential regulators of apoptosis [29, 34], whereas *CCL24* was increased in broncho-alveolar lavage (BAL) of patients with stage 3 sarcoidosis compared to patients with Lo¨ fgren syndrome [58] and *IL23R* has been implicated in the formation of granulomas [38]. Concerning the latter, it is worth noting that previous attempts to use ustekinumab, an anti IL-12 and IL-23 monoclonal antibody, have been unsuccessful in the management of sarcoidosis [42].

Of great interest, *PLCL1*, *PPARG*, *CD19* have been involved in sarcoidosis through functional studies [49–56]. These factors are probably the most promising for further investigations as possible targets for therapeutic strategies.

Finally, at least 8 additional genes have been implicated in immune regulatory response, whereas no clear evidence linking *ACOXL*, *MAT1A*, or *MACROD1* to sarcoidosis or immune (dys)regulation was found from the available literature. Further studies are required to define a possible involvement of these factors in the pathogenesis of the disease, which if confirmed would pave the way to previously unforeseen clues about the pathogenesis and therapeutic options for sarcoidosis.

Among the limitations of this study, all those stemming from a meta-analysis of public summary statistics must be taken into account: in particular, as access to individual-level data was not possible, further delving into genomic analysis (e.g. through validation of polygenic risk scores or Mendelian randomisation) was not feasible. Furthermore, it is important to note that the available phenotype information for patients only stated whether they were affected by sarcoidosis, but did not specify other clinically relevant details such as the targeted organs or phenotype severity.

Finally, although slightly different results can be obtained depending on the choice of the technical parameter to use for meta-analysis and functional annotation, the core results of the study have been robust even after repeating calculations with different specifics. Additionally, although further analysis (e.g. chromatin analysis) could be implemented through the available platforms, we put our best efforts into prioritising the most relevant information, with a focus on clinical interpretation of the results.

## Conclusion

We present a GWAS meta-analysis for sarcoidosis in European and African ancestries, obtained from the most recent releases of publicly available summary statistics of the FinnGen, MVP, pan-UKBB, and BioBank Japan projects. Nineteen and two risk loci were significantly associated with sarcoidosis for European and African ancestries, respectively. Mild-moderate genetic heritability was observed for both

ancestries. From comparison between European and multi-ancestry meta-analysis, novel risk loci private to EUR ancestry could be hypothesised; on the other hand, trending loci in EUR ancestry confirmed their significance when aggregating ancestries. Crucially, more efforts are needed to collect data from African and East Asian ancestries, as the current sample sizes in both control and - especially - cases are likely insufficient to obtain novel ancestry-specific information through meta-analysis.

Critical revision and comparison with the available literature has highlighted several genes as putatively associated with the pathogenesis of sarcoidosis, thus paving the way to further investigation as possible disease-altering factors or therapeutic targets. Although further studies are warranted, epigenetic alterations may contribute to the risk of developing the phenotype.

We hope that the interpretation of these new data will contribute to a more precise understanding of the etiology and pathophysiology of sarcoidosis at the molecular level, with the potential to aid in the development of more personalized approaches for diagnosis, prognosis, and treatment.

## Electronic supplementary material

Below is the link to the electronic supplementary material.

Supplementary material 1

## Data Availability

R markdown sheets are available in Supplementary Files. Further material can be made available upon reasonable request to the Authors.

## Abbreviations

1000 G: 1000 genomes (Project Phase 3 reference panel)
AFR: African (ancestry)
BAL: broncho-alveolar lavage
BBJ: Biobank Japan
CADD: Combined Annotation-Dependent Depletion
GTEx: Genotype- Tissue Expression (progect)
EAS: East Asian (ancestry)
eQTL: expression quantitative trait locus/i
EUR: European (ancestry)
FUMA: Functional Mapping and Annotation (tool)
GSEA: gene-set enrichment analysis
GWAS: genome-wide association study
LD(SC): linkage disequilibrium (score)
MHC: major histocompatibility complex
MVP: Million Veteran Program
SNP: single nucleotide polymorphism
TEA: tissue expression analysis
TCR: T cell receptor
UKBB: UK Biobank
WES: whole exome sequencing
WGS: whole-genome sequencing
